# Altered Gut Microbiota Taxonomic Compositions of Patients With Sepsis in a Pediatric Intensive Care Unit

**DOI:** 10.3389/fped.2021.645060

**Published:** 2021-04-07

**Authors:** Jing Liu, Mingbang Wang, Weiming Chen, Jian Ma, Yi Peng, Mingzhi Zhang, Chuanqing Wang, Gangfeng Yan, Guoping Lu

**Affiliations:** ^1^Pediatric Intensive Care Unit, Children's Hospital of Fudan University, National Children's Medical Center, Shanghai, China; ^2^Shanghai Key Laboratory of Birth Defects, Division of Neonatology, Children's Hospital of Fudan University, National Children's Medical Center, Shanghai, China; ^3^International Clinic, Children's Hospital of Fudan University, National Children's Medical Center, Shanghai, China

**Keywords:** sepsis, gut microbiota, 16S rRNA gene, *Enterococcus*, short-chain fatty acids

## Abstract

**Background:** The gut is thought to play an important role in the pathogenesis of sepsis. Changes in the gut microbiota are closely related to the occurrence and development of human diseases, but few studies have focused on taxonomic composition of gut microbiota in septic patients. Knowledge of changes in the gut microbiota is a key issue in intensive care. Clinicians must understand how an altered gut microbiota affects the susceptibility and prognosis of septic patients.

**Measurements and Main Results:** In the single-center case control study, 20 septic patients and 20 healthy children were recruited. The taxonomic composition of gut microbiota was determined *via* 16S rRNA gene sequencing. Gut microbiota diversity in children with sepsis was significantly reduced compared with that in healthy children. The taxonomic composition of gut microbiota can effectively distinguish children with sepsis from healthy children. Thirteen taxa of gut microbiota were significantly increased in the guts of children with sepsis compared with those of healthy children. The increased abundances of Enterococcaceae, *Enterococcus*, and *Enterococcus durans* in gut of septic patients were significantly positively correlated with blood inflammation indicators CRP and WBC. The abundances of seven bacteria were significantly decreased in the guts of septic children compared with those of healthy children. The decreased abundance of Bifidobacteriales in gut of septic patients is significantly negatively correlated with blood inflammation index WBC. A machine-learning classifier was built for distinguishing sepsis and achieved the AUC value of 81.25%. It shows that the composition of gut microbiota has certain potential for diagnosis of sepsis.

**Conclusions:** Gut microbiota alterations in septic patients exhibit proliferation of opportunistic pathogenic bacteria, the massive reduction of the commensal flora, and the significant decrease in the diversity of the gut microbiota. Dysbiosis may also account for some changes in the inflammation indexes.

## At a Glance Commentary

### Scientific Knowledge on the Subject

The gut is thought to play an important role in the pathogenesis of sepsis. Knowledge of changes in the gut microbiota has been identified as a key issue in the field of intensive care. It is important to understand how changes in the gut microbiota affect the susceptibility and prognosis of septic patients.

### What This Study Adds to the Field

Gut microbiota changes in patients with sepsis include significant increases in harmful bacteria, such as *Enterococcaceae*, and significant reductions in bacteria, such as Lachnospiraceae, Ruminococcaceae, Peptostreptococcaceae, and Acidaminococcaceae, which are short-chain fatty-acid-producing bacteria. These bacteria are potential biomarkers of sepsis.

## Introduction

Sepsis is a life-threatening organ failure caused by a host's dysfunctional response to infection. Considering the high morbidity and mortality of sepsis, it is a public health problem that affects ~1.2 million children worldwide each year (1). Sepsis is the most common cause of death in hospitalized patients, especially in intensive care units (ICUs), with a global mortality rate approaching 25% (2). Assessing organ failure in septic patients focuses primarily on the respiratory, cardiovascular, hepatic, renal, nervous, and circulatory systems. Although the gut is considered to play an important role in the pathogenesis of sepsis, symptoms of gut failure are non-specific and usually not evaluated (3).

The human gut microbiota is a complex ecosystem of trillions of bacteria, constituting the largest and most heterogeneous community in the gastrointestinal tract (4). Using culture-independent 16S rRNA and shotgun metagenomic sequencing methods, increasing studies have begun to clarify the correlation between the gut microbiota and human diseases. For example, reduction of the gut microbiota diversity or dysbiosis is associated with obesity, *Clostridium difficile* infection (5, 6), and increased mortality in patients after allogeneic hematopoietic stem cell transplantation (7).

Few studies have used high-throughput sequencing techniques to study the taxonomic composition of gut microbiota in patients with sepsis. Two small studies involving ICU patients (8, 9) revealed significant alterations in the taxonomic composition of gut microbiota of patients with sepsis. The guts of septic patients are dominated by individual bacterial species, including several pathogenic, and antibiotic-resistant species, such as *Clostridium* and *Enterococcus*. The guts of septic patients are also lacking important bacterial genera, including *Faecalibacterium, Prevotella*, and *Blautia*, and the family Ruminococcaceae, which produce short-chain fatty acids (SCFAs) (10). These SCFAs are significantly reduced in critically ill patients, which can adversely affect the intestinal integrity and systemic immunity in septic patients (11, 12). The disappearance of *Faecalibacterium prausnitzii*, which has an anti-inflammatory effect, may further promote an adverse inflammatory state in the intestines (13).

Understanding the changes in the gut microbiota during sepsis is a key issue in critical care medicine, and clinicians must understand how changes in the gut microbiota affect the susceptibility and prognosis of septic patients. The aim of our study was to find the changes of gut microbiota in septic patients and whether if affects the susceptibility and prognosis of septic patients.

## Methods

### Participants and Clinical Evaluation

Patients with sepsis were recruited from the pediatric intensive care unit (PICU) in Children's Hospital of Fudan University from January 2018 to June 2018. Sepsis was diagnosed according to the International Consensus Conference on Pediatric Sepsis (14, 15). Twenty children with sepsis in a PICU were recruited: 10 with confirmed pathogen infections and 10 without confirmed pathogen infections. Twenty healthy children (HC) were recruited as controls. Fecal samples and clinical information were collected, and the association of the taxonomic composition of gut microbiota with sepsis and clinical phenotype was assessed. The clinical evaluation included non-specific blood examinations, with white blood cell, C-reactive protein, procalcitonin, and platelet counts. HC were those who attended health checkups in the health-care department of our hospital and were recruited as controls. All septic patients have undergone antibiotic treatment, and the sampling time is after treatment. The Ethics Committee of the Children's Hospital of Fudan University approved the study, which was performed in accordance with the Declaration of Helsinki. A signed consent was obtained by all patients' parents. Feces were collected, immediately stored in a freezer at −20°C, then transferred to a freezer at −80°C on either the same day or the next.

### 16S rDNA Gene Sequencing

Genomic DNA was extracted using the StoolGen fecal DNA extraction kit (CW Biotech, Beijing, China). The extracted DNA was diluted to 1 ng/μl and used as the template DNA. The primers 515F (5'-GTG CCA GCM GCC GCG GTA A-3') and 806R (5'-GGA CTA CNN GGG TAT CTA AT-3') were used to amplify the V4 region of the 16S rDNA gene for the polymerase chain reaction (PCR) to ensure amplification efficiency and accuracy. PCR was performed using Phusion® High-Fidelity PCR Master Mix (New England Biolabs, Ipswich, MA, USA). The PCR product was recovered using a gel recovery kit (Qiagen, Hilden, Germany), and the libraries were constructed using TruSeq® DNA PCR-Free Sample Preparation Kit (Illumina, San Diego, CA, USA) and sequenced using the HiSeq2500 System (model PE250, Illumina).

### Gut Microbiota Taxonomic Profiling

The gut microbiota taxonomic profiling was performed as previously described (16). Briefly, the barcode and primer sequences were truncated using FLASH (17) to obtain the raw reads, and then the raw reads were subjected to quality control using QIIME2 (18). Chimera sequences were removed using UCHIME (19) to obtain clean reads, which were clustered into operational taxonomic units (OTUs) using UPARSE (20). The OTUs were annotated in the Mothur (21) and SILVA databases (22) to obtain the gut microbiota taxonomic profiling data. The phylogenetic relationships of all OTUs were obtained using MUSCLE (23). Taxonomic profiling data were normalized according to the sample with the fewest data for further microbiome analysis.

### Microbiome Analysis

The common/exclusive OTUs among the groups are shown in a Venn diagram. To assess whether the sample size was sufficient, a species accumulation boxplot was used to assess whether the species richness increased when the sample size increased. The gut microbiota diversity indexes were completed using the vegan package's diversity function and displayed using R's boxplot function. Differences in the gut microbiota diversity were determined *via* the Wilcoxon rank sum test using R's wilcox.test function. To determine whether the gut microbiota can be used to distinguish between groups, Non-metric multidimensional scaling (NMDS) was performed using vegan, stats, and ggplot2 package in R (version 3.6.3). Non-parametric multivariate analysis of variance based on Bray-Curtis distance or permutation multivariate analysis of variance (PERMANOVA) (24, 25) were performed using R's adonis function to assess whether the clinical phenotype significantly affected the taxonomic composition of gut microbiota. The values ^*^p < 0.05, ^**^p < 0.01, and ^***^p < 0.001 were considered statistically significant.

### Microbiomewide Association Analysis

Taxonomic composition of gut microbiota associated with sepsis were identified as per our previous studies (26–28). The criteria for screening enriched taxonomic composition of gut microbiota for the disease or control groups were mean relative abundance > 0.1%, coverage > 80%, fdr ≤ 0.05 for both deseq2 and Wilxon rank sum test, and logfoldchange >0.58 for deseq2 test.

### Regression Analysis

statannot package (version 0.2.3) was used for analysis of differences between groups, and the selected test method was Mann-Whitney, and the stats function of the spicy package (version 1.5.0) was used for linear regression analysis.

### ROC Analysis

Machine-learning methods RandomForestClassifier function in the scikit learn package (version 0.23.1) was used to determine to build a classifier that can be used for disease classification, and finally roc_curve in scikit learn package was used to plot the receiver operating characteristic (ROC) curve and area under the curve (AUC) value of the classifier.

## Results

Forty samples were included in the present study. Table 1 summarizes the clinical information statistics (see Supplementary Table 1 for details). Twenty children had sepsis, including 10 with confirmed pathogenic microorganismal infections (sepsis with infection, SI) and 10 with no identified pathogenic microorganismal infection (sepsis without infection, SE). Twenty HC were included as controls. Supplementary Figure 1 shows the overall analysis process.

**Table 1:** Summary of clinical information.

**Characteristics**	**HC (*n =* 20)**	**SI (*n =* 10)**	**SE (*n =* 10)**
Male gender, Percentage	85.0%	80.0%	80.0%
Age, year old, mean ± SD	6.35 ± 2.0	3.40 ± 1.43	3.80 ± 2.30
WBC, 10^9^/L, mean ± SD	NA	14.57 ± 3.88	12.8 ± 4.55
CRP, mg/L, mean ± SD	NA	37.0 ± 38.93	34.30 ± 26.70
PCT, ng/ml, mean ± SD	NA	10.60 ± 31.42	0.71 ± 1.10

*CRP, C-reactive protein; HC, healthy children; SE, sepsis without confirmed pathogens; PCT, procalcitonin; SI, sepsis with confirmed pathogens infection; WBC, whole blood cell; SD, Standard Deviation*.

### 16S rRNA Analysis of the Taxonomic Composition of Gut Microbiota in Septic Patients

To determine whether the sample size was sufficient, a species accumulation boxplot was constructed. Supplementary Figure 2a shows that when the sample size was >20, the occurrence rate of new OTUs (new species) decreased under continuous sampling, and when the sample size was >30, the OTUs approached saturation. Thus, our sample size was sufficient for data analysis. To determine the common and unique OTUs between the groups, the OTU cluster analysis results were analyzed *via* Venn diagram (Supplementary Figure 2b). The number of common OTUs between the HC vs. SE, HC, vs. SI, and SE vs. SI groups were 755, 776, and 687, respectively, and 669 OTUs were shared among the three groups, which were mostly OTUs shared by two of the groups. Supplementary Figure 2c shows the taxonomic compositions of the gut microbiota at the phylum level for each sample. The major four phyla of the human gut microbiota are Bacteroidetes, Firmicutes, Proteobacteria, and Actinobacteria. Stool samples from the HC demonstrated a dominance of Firmicutes and Bacteroidetes, whereas Proteobacteria or Firmicutes were dominant in septic patients. The abundance of Proteobacteria in patients with sepsis was significantly higher than that in the HC.

### Taxonomic Composition of Gut Microbiota Distinguished Septic Patients From Healthy Children

**Figure 1:**
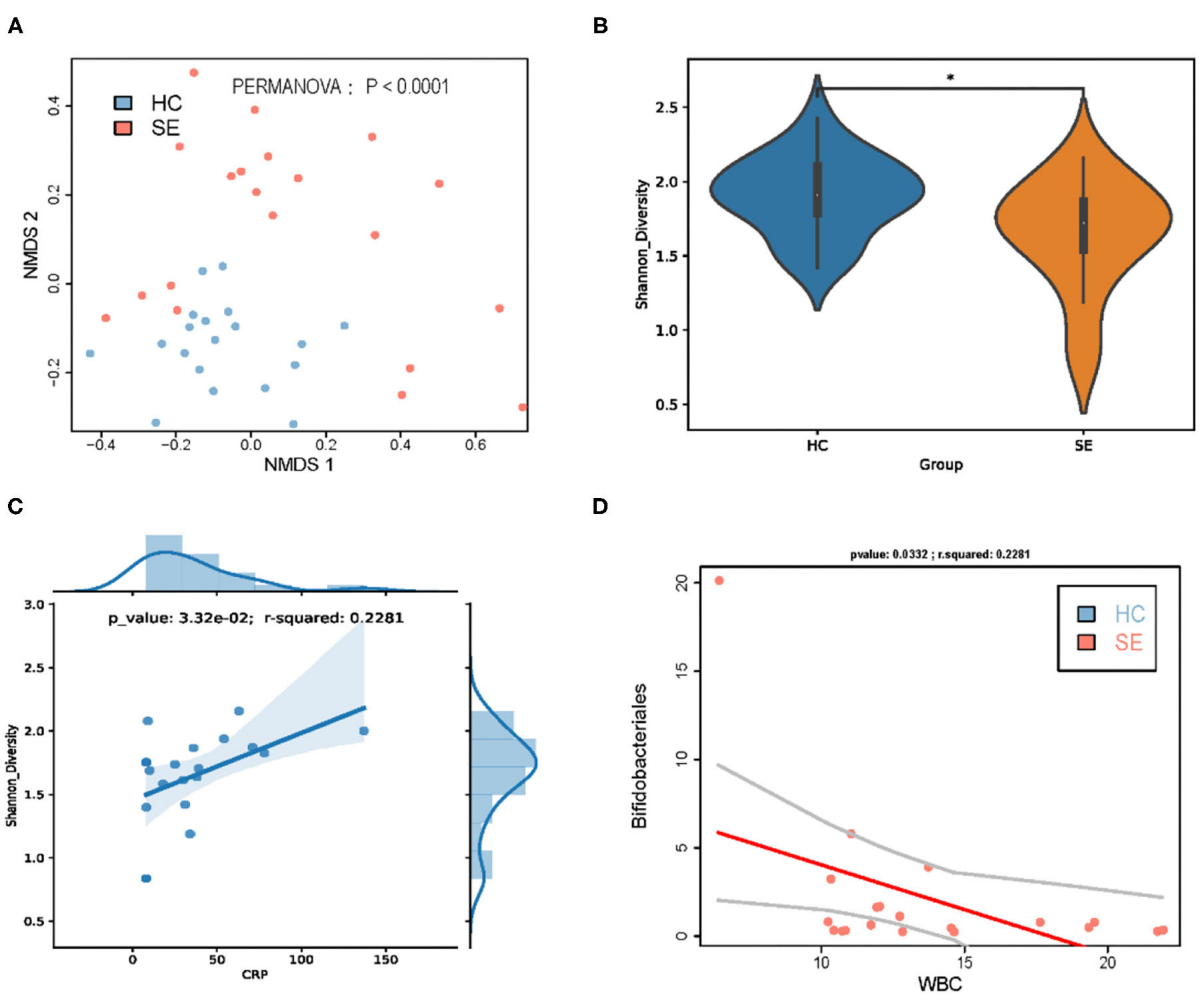
Gut microbiota taxa distinguished sepsis patients from healthy children. **(A)** NMDS of taxonomic composition of gut microbiota of HC vs. SE. **(B)** Gut microbiota Shannon diversity of HC vs. SE. **(C)** In sepsis, gut microbiota Shannon diversity was positively correlated with CRP; **(D)** In sepsis, the gut abundance of Bifidobacteriales was negatively correlated with WBC. *p < 0.05.

To further evaluate whether the intestinal flora can be used to distinguish between patients with sepsis and HC, NMDS was performed, and two principal coordinates are shown in Figure 1A. The NMDS showed that samples from the sepsis patients were distanced far from those of the HC and could be clearly distinguished. The results were consistent with the results of the PERMANOVA, that is, sepsis significantly affected the taxonomic composition of gut microbiota (*P* < 0.0001). To determine whether the taxonomic composition of gut microbiota can be used to distinguish different groups, the ecological diversity indexes of the samples were calculated and compared between the groups. The Shannon indexes of the SE group were significantly lower than that of the HC group (Figure 1B). At the same time,we found that the gut shannon diversity index in children with sepsis was positively correlated with the increase in blood CRP (Figure 1C).

### Taxonomic Composition of Gut Microbiota Is Associated With Sepsis and Clinical Indexes

To further identify the taxonomic composition of gut microbiota associated with sepsis, a microbiomwide association study was performed, and 20 gut microbiota taxa had significantly different abundances in SE vs. HC groups, 13 of which were taxa with significantly increased abundances in patients with sepsis compared with the HC group: phylum Proteobacteria, classes Bacilli and Gammaproteobacteria, orders Enterobacteriales, Pseudomonadales, and Lactobacillales, families Enterococcaceae, Enterobacteriaceae, and Moraxellaceae, genus *Enterococcus, Clostridium innocuum_group*, and *Acinetobacter*, and species *Enterococcus durans*. Seven taxa had significantly decreased abundances in patients with sepsis compared with those in the HC: orders Bifidobacteriales and Selenomonadales, family Acidaminococcaceae, genus *Erysipelotrichaceae UCG-003* and *Dialister*, species *Dorea longicatena* and *Ruminococcus sp. 5_1_39BFAA* (Supplementary Table 2).

**Figure 2:**
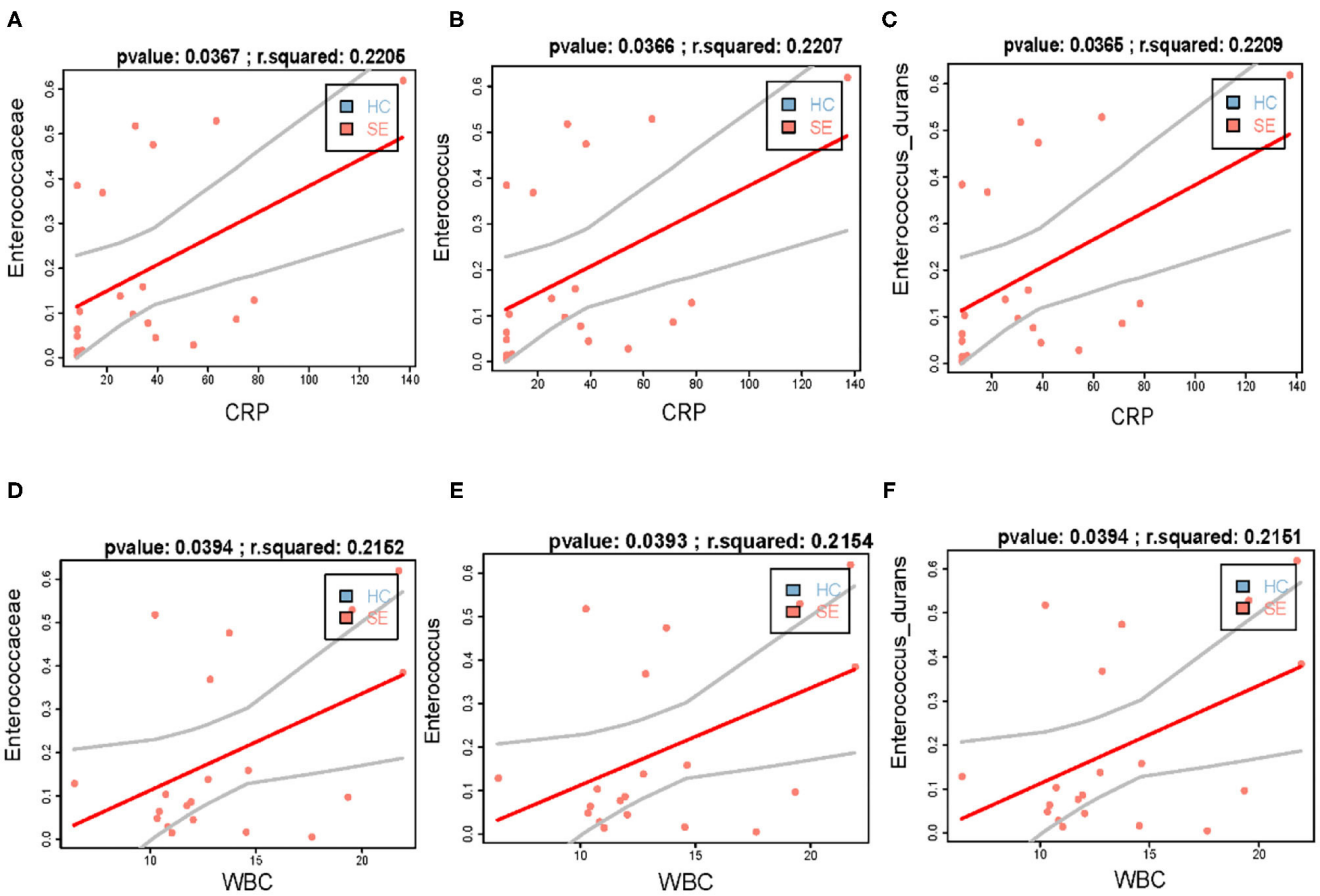
The increase in the abundance of *Enterococcaceae* bacteria in gut of patients with sepsis is positively correlated with the increase in blood indicators WBC and CRP. **(A–C)** the increased abundance of gut *Enterococcaceae* **(A)**, *Enterococcus* **(B)**, and *Enterococcus durans* **(C)** is positively correlated with WBC, respectively; **(D–F)** the increased abundance of gut *Enterococcaceae* **(D)**, *Enterococcus* **(E)**, and *Enterococcus durans* **(F)** is positively correlated with CRP, respectively.

It's worth noting that we found that the increase in the abundance of family Enterococcaceae bacteria, including family Enterococcaceae, genus *Enterococcus*, and *Enterococcus durans* in gut of patients with sepsis is positively correlated with the increase in blood indicators WBC and CRP (Figure 2). At the same time, we found that Acidaminococcaceae and *Dorea longicatena*, which were significantly decreased bacteria in gut of septic patients compared with HC, were significantly positively correlated with Shannon diversity (Supplementary Figures 3a,b), and that *Erysipelotrichaceae UCG-003* and *Dialister*, which were also significantly decreased bacteria in gut of septic patients compared with HC, was not affected by infection (Supplementary Figures 3c,d).

### Taxonomic Composition of Gut Microbiota Are Potential Markers for the Diagnosis of Sepsis

**Figure 3:**
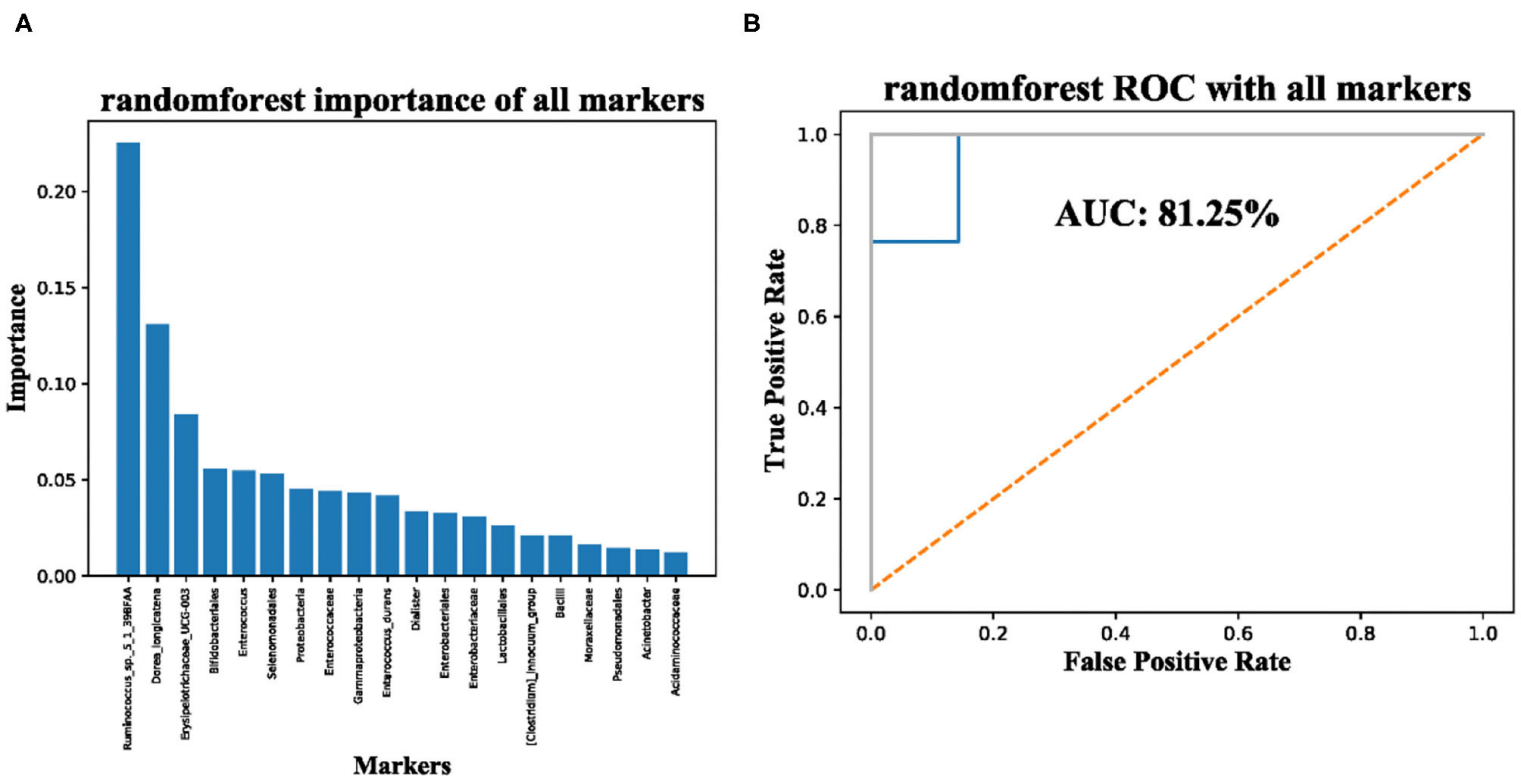
Significantly enriched taxa in the guts of sepsis patients are potential makers for diagnosis of sepsis. **(A)** importance of significantly enriched taxa; **(B)** AUC of significantly enriched taxa used for sepsis diagnosis.

Finally, we used the machine-learning method RandomForest to evaluate whether the 20 taxonomic composition of gut microbiota has the potential to be used as a marker for the diagnosis of sepsis. The importance is shown in Figure 3A and the ROC scores achieved 81.25% (Figure 3B), Although in-depth research is needed, our results suggest that the taxonomic composition of gut microbiota is a potential biomarker.

## Discussion

The gut microbiota plays an important role in maintaining intestinal barrier function and regulating the innate and adaptive immune systems (11). Culture-independent methods, such as 16S rRNA gene and shotgun metagenomic sequencing methods, have provided increasing evidence to suggest that the gut microbiota is an important player in the pathophysiology of sepsis (8, 9). Sepsis affects the taxonomic composition of gut microbiota. Severe illnesses can interfere with the taxonomic composition of gut microbiota, likely due to the devastating effects of the illness as well as the intervention during clinical care because patients with sepsis are usually treated with at least two antimicrobial agents (29). Furthermore, factors such as hypoxic injury, inflammation, intestinal dysfunction, destruction of epithelial integrity, changes in intraluminal pH, vasopressor therapy, proton-pump inhibitors, opioids, and parenteral or enteral feeding, are considered key potential disruptive factors in the microbiome (30).

We found that the abundance of family Enterococcaceae, genus *Enterococcus* and species *Enterococcus durans* in the feces of children with sepsis were significantly higher than that of the HC; family Enterococcaceae bacteria are important pathogen of nosocomial infections, they can enter the bloodstream through body surfaces or infected sites and spread, causing severe consequences, such as bacteremia, a type of sepsis (31). Rogers et al. found that the abundance of *Enterococcus* in the intestine of PICU children was significantly higher than that of HC (32). Ryu et al. found that *Enterococcus durans* are mainly derived from the biliary or urinary tracts (33). At the same time, we found that the significant increase of Enterobacteriaceae bacteria in septic children is positively correlated with blood inflammation indicators WBC and CRP.

We found that the gut microbiota Shannon diversity in patients with sepsis was significantly reduced compared with that in HC, which is consistent with the result of Rogers et al. (32). The guts of patients with sepsis lack key bacteria that represent important components of the microbiota of healthy individuals. For example, we found that the abundances of family Acidaminococcaceae was significantly reduced in patients with sepsis compared with those of HC. Studies have shown that family Acidaminococcaceae bacteria are SCFA-producing bacteria (10), which can accumulate acetic acid and butyric acid in a medium containing amino acids, studies have showed that acetic acid and butyric acid are important SCFAs that can regulate the differentiation and expansion of several T-cell types to form a complete mucosal immune system (11, 12, 34). O'Keefe et al. found that SCFAs were significantly lower in critically ill patients than in HC (35). We also found that the abundances of order Bifidobacteriales was significantly reduced in patients with sepsis compared with those of HC. Order Bifidobacteriales bacteria, which are important probiotics of human gut microbiota, are reported to have anti-inflammatory properties (34). It is worth noting that we found that the decrease of gut Bifidobacteriales abundance in septic patients was significantly negatively correlated with WBC (Figure 1D) and that the significant decrease in the abundance of *Erysipelotrichaceae UCG-003* and *Dorea longicatena* in the gut of septic patients was significantly positively correlated with the decrease in the Shannon diversity index of the gut microbiota.

Finally, we use machine-learning methods and sepsis-related intestinal bacteria to construct a classifier for distinguishing sepsis, and the AUC value can reach 81.25%; although further verification is needed, it shows that the composition of gut microbiota has certain potential for diagnosis of sepsis. In addition, Giordano et al. found that probiotics may play a promising role in modifying the intestinal microbiota of patients with STEC (Shigatoxin-producing *E. coli*) gastroenteritis, thus avoiding the onset or, at least, reducing the severity of Hemolytic Uremic Syndrome (36). Managing dysbiosis and manipulating the microbial environment with probiotic supplementations is a promising research field for promoting health and preventing diseases also in term and preterm neonates (37). Therefore, targeting the gut microbiota may represent a new potential therapeutic strategy in septic children. The strengths of our study are that we focus on a serious disease in children and describe the changes of intestinal microbiota through non-invasive methods, with the aim to further explore new treatment options.

This study also had some limitations. As the gut SCFA-producing bacteria of septic children are significantly reduced, it is necessary to check whether the gut and blood SCFA metabolites have changed to further verify the correlation between SCFA-producing bacteria and sepsis. Furthermore, gut bacteria associated with sepsis require further validation to confirm whether these gut bacteria can be specific biomarkers for sepsis. Finally, researchers must determine whether the gut bacteria are significantly altered before and after intervention to further evaluate whether the bacteria can be used as therapeutically relevant biomarkers.

In conclusion, our research suggests the dysbiosis of the gut microbiota in children with sepsis. Gut microbiota alteration in septic patients exhibit proliferation of opportunistic pathogenic bacteria, the massive reduction of the commensal flora, and the significant decrease in the diversity of the gut microbiota. Dysbiosis may also account for some changes in the inflammation indexes.

## Data Availability Statement

The sequencing data has been deposited into a publicly accessible repository: https://db.cngb.org/search/project/CNP0001554/.

## Ethics Statement

The studies involving human participants were reviewed and approved by Children's Hospital of Fudan University. Written informed consent to participate in this study was provided by the participants' legal guardian/next of kin.

## Author Contributions

JL and MW conceptualized and designed the study and wrote the article. GY and GL reviewed and revised the manuscript. WC, JM, YP, MZ, and CW coordinated and supervised data collection. MW carried out the analyses and interpreted the results. All authors contributed to manuscript revision and read and approved the submitted version.

## Conflict of Interest

The authors declare that the research was conducted in the absence of any commercial or financial relationships that could be construed as a potential conflict of interest.
