# Supplementary material for: Altered Gut Microbiota Taxonomic Compositions of Patients With Sepsis in a Pediatric Intensive Care Unit
Source: Front Pediatr. 2021 Apr 7;9:645060. doi: 10.3389/fped.2021.645060 (PMC8058355; doi:10.3389/fped.2021.645060)
Supplement: Supplementary file 1 [file Data_Sheet_1.docx]

**Supplementary Figures**


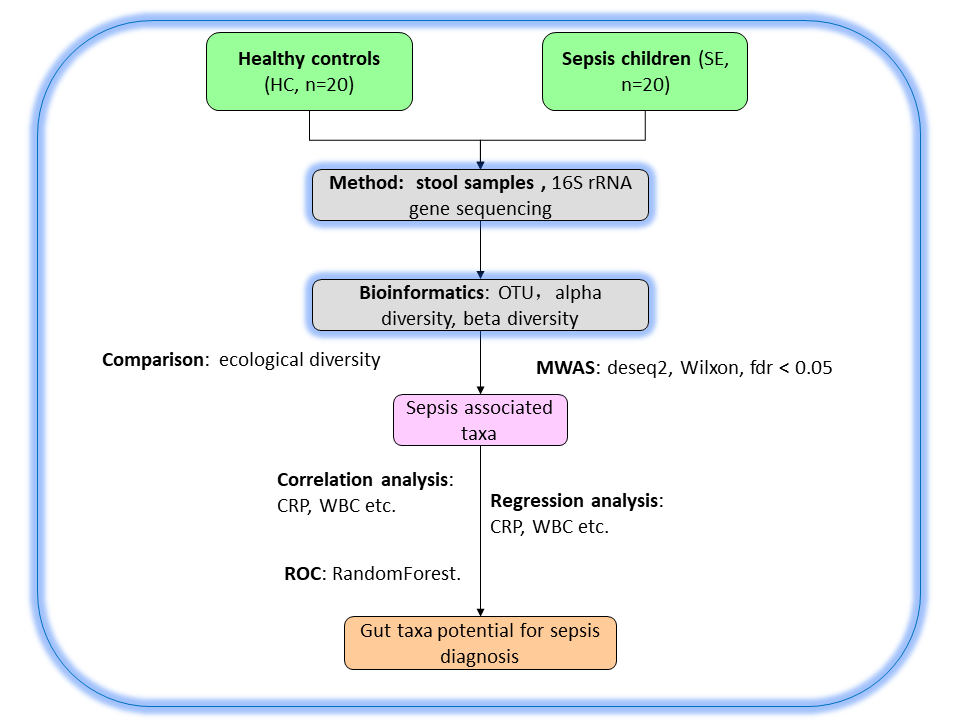


**Supplementary Figure 1. Flowchart of the study design.** Stool samples from 20 children with sepsis (SE) and 20 healthy children (HC) were included in the study. 16S rRNA gene sequencing and microbiome analysis was performed, including OTU identification, alpha and beta diversity comparison, and differential gut microbiota analysis. To determine the sepsis-associated taxa in gut microbiota, the deseq2 and Wilcoxon rank sum test was used to determine significant differences in the SE vs HC. Correlation and regression analysis were performed between the sepsis-associated taxa in gut microbiota and clinical phenotypes such as serum CRP and WBC levels, ROC performed using machine learning methods RandomForest to determine potential gut taxa for sepsis diagnosis.


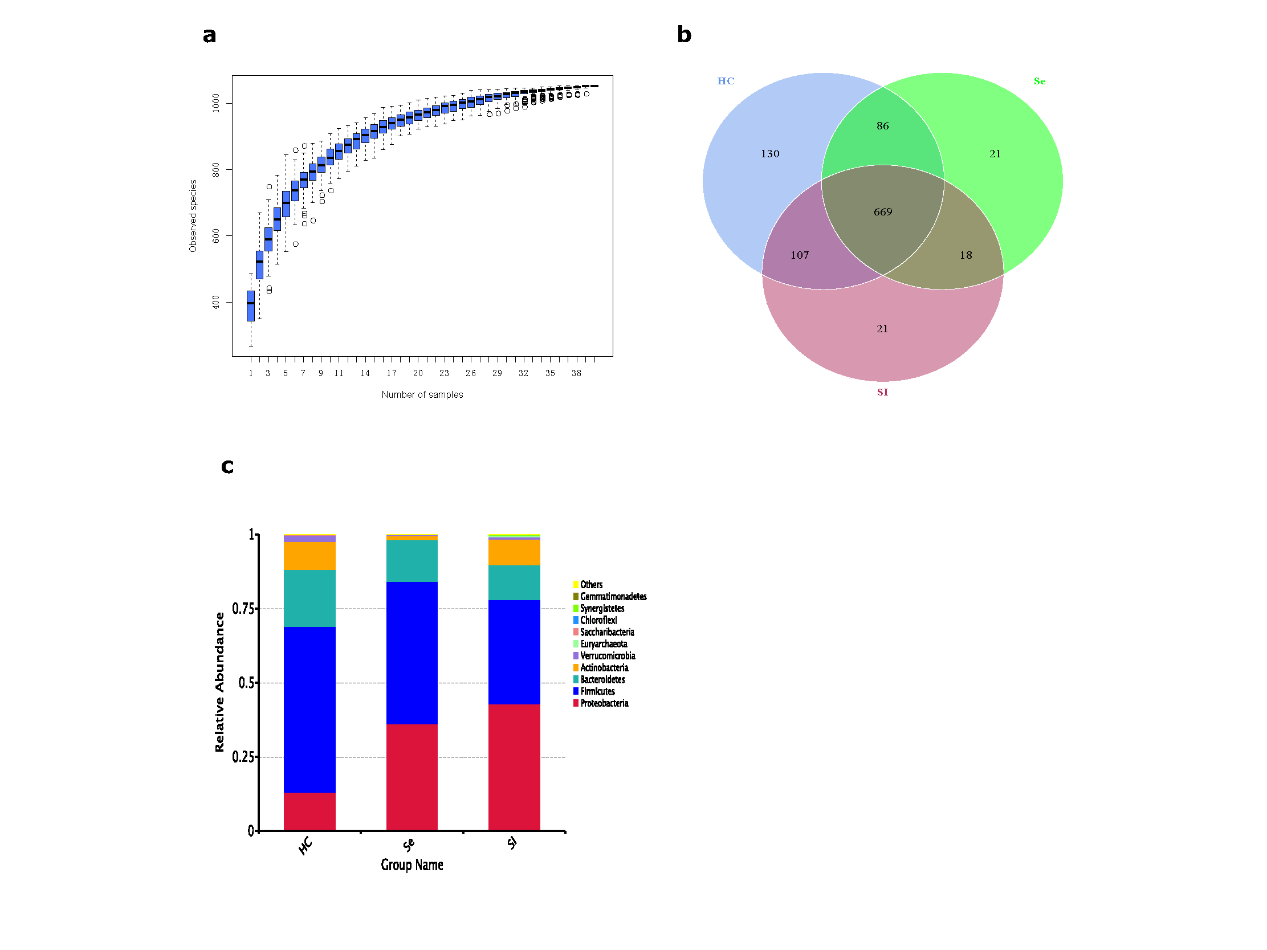


**Supplementary Figure 2. Gut microbiota taxa in patients with sepsis**. a, Species accumulation boxplot showing that when the sample size was greater than 20, the rate of occurrence of new OTUs (new species) decreased under continuous sampling, and when the sample size was greater than 30, the number of OTUs approached saturation, indicating that the sample size was sufficient for analysis. The x-axis is the sample size; the y-axis is the number of OTUs. b, Venn diagram based on the OTUs; each circle in the figure represents a set of samples, the number of overlapping circles represents the number of OTUs shared between groups, and the number without overlap represents the number of unique OTUs in the group. c, Taxonomic composition of gut microbiota at the phylum level; the top 10 phyla were used.

**
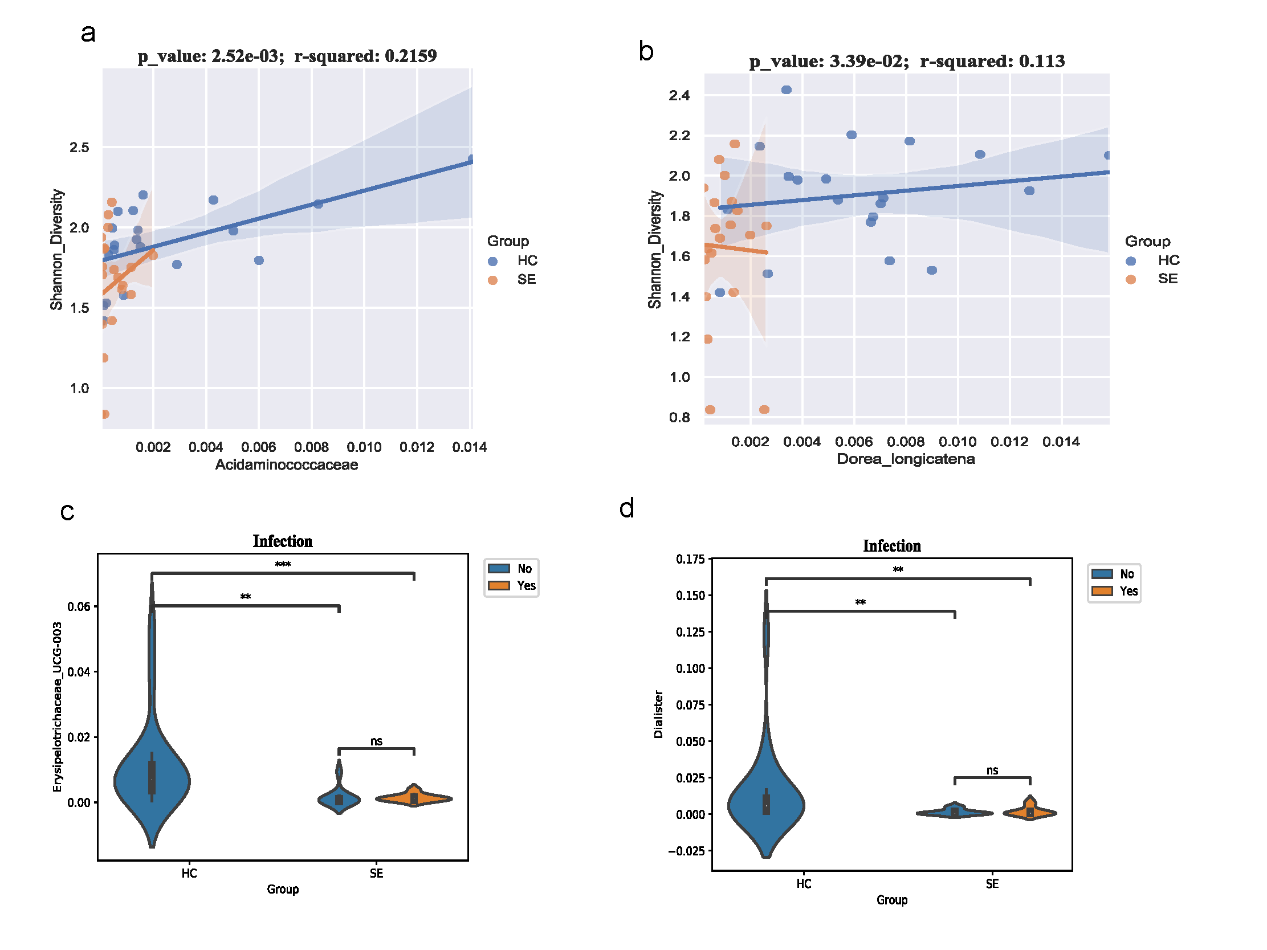
**

**Supplementary Figure 3.** Sepsis decreased taxa were associated with Shannon diversity and not affected by infection. **a-b**, Sepsis decreased taxa Acidaminococcaceae (a) and *Dorea longicatena* (b) were significantly positively correlated with Shannon diversity; **c-d**, Sepsis decreased taxa *Erysipelotrichaceae UCG-003* (c) and *Dialister* (d) were not affected by infection.
